# Deficiency of Cbfβ in articular cartilage leads to osteoarthritis-like phenotype through Hippo/Yap, TGFβ, and Wnt/β-catenin signaling pathways

**DOI:** 10.7150/ijbs.90250

**Published:** 2024-03-11

**Authors:** Yan Zhang, Huiwen Chen, Jinjin Wu, Abigail McVicar, Yilin Chen, Jiacan Su, Yi-Ping Li, Wei Chen

**Affiliations:** 1 Department of Pathology, University of Alabama at Birmingham, Birmingham, AL 35294, USA.; 2 Key Laboratory of Biomedical Information Engineering of Ministry of Education, Biomedical Informatics and Genomics Center, School of Life Science and Technology, Xi'an Jiaotong University, Shaanxi, Xi'an 710049, P.R. China.; 3 Division in Cellular and Molecular Medicine, Department of Pathology and Laboratory Medicine, Tulane University School of Medicine, Tulane University, New Orleans, 70112, USA.; 4 Institute of Translational Medicine, Shanghai University, Shanghai, P.R. China.

**Keywords:** Osteoarthritis, Cbfβ, Wnt signaling, Hippo/YAP signaling, TGFβ signaling

## Abstract

Osteoarthritis (OA) is the most prevalent degenerative joint disorder, causing physical impairments among the elderly. Core binding factor subunit β (Cbfβ) has a critical role in bone homeostasis and cartilage development. However, the function and mechanism of Cbfβ in articular cartilage and OA remains unclear. We found that Cbfβ^f/f^Aggrecan-CreER^T^ mice with Cbfβ-deficiency in articular cartilage developed a spontaneous osteoarthritis-like phenotype with articular cartilage degradation. Immunofluorescence staining showed that Cbfβ^f/f^Aggrecan-CreER^T^ mice exhibited a significant increase in the expression of articular cartilage degradation markers and inflammatory markers in the knee joints. RNA-sequencing analysis demonstrated that Cbfβ orchestrated Hippo/Yap, TGFβ/Smad, and Wnt/β-catenin signaling pathways in articular cartilage, and Cbfβ deficiency resulted in the abnormal expression of downstream genes involved in maintaining articular cartilage homeostasis. Immunofluorescence staining results showed Cbfβ deficiency significantly increased active β-catenin and TCF4 expression while reducing Yap, TGFβ1, and p-Smad 2/3 expression. Western blot and qPCR validated gene expression changes in hip articular cartilage of Cbfβ-deficient mice. Our results demonstrate that deficiency of Cbfβ in articular cartilage leads to an OA-like phenotype via affecting Hippo/Yap, TGFβ, and Wnt/β-catenin signaling pathways, disrupting articular cartilage homeostasis and leading to the pathological process of OA in mice. Our results indicate that targeting Cbfβ may be a potential therapeutic target for the design of novel and effective treatments for OA.

## Introduction

Osteoarthritis (OA) is a widely prevalent joint degeneration disease, a primary source of pain and disability due to chronic inflammation, frequently resulting in significant movement restrictions and physical impairments in individuals over 55 years old 1, 2. Moreover, OA involves damage to multiple types of tissue 3, however the direct causes of joint degeneration in OA remain unclear. OA is characterized by cartilage degradation, subchondral bone sclerosis or bone loss, osteophyte formation and high expression of cartilage degradation enzymes matrix metalloproteinases (MMPs) and aggrecanases (ADAMTSs) 4. Osteophyte formation is one of the classic outcomes of progressive OA, characterized by cartilage degeneration and endochondral ossification 5, 6. After the cartilage is destroyed, the perichondrium hyperplasia of new bone, after ossification forms osteophytes, which is a compensatory reaction in OA pathogenesis 5. Most studies have examined the later stages of the disease, leaving the initial factors that cause OA largely unknown 7. Consequently, current treatments for OA are often palliative and in many cases require joint replacement 8, which is costly and functionally finite. Transcription factors play the role of key regulatory factors in cells, controlling the process of disease occurrence, and their dysfunction is a major pathogenic factor 9. Fully understanding how transcription factors regulate bone formation and maintain bone homeostasis is vital for developing treatments for OA.

Core binding factors (Cbfs) are heterodimeric transcription factors consisting of Cbf-beta (Cbfβ) subunits and Cbf-alpha (Cbfα) subunits 10, 11. Cbfβ/Cbfα heterodimeric transcription complexes are key players in many developmental processes, including chondrocyte proliferation, commitment, and differentiation, as well as osteoblast differentiation 10-17. We previously found that one of the genes encoded by the Cbfα subunits, Cbfα2 (also known as Runt-related transcription factor 1 (Runx1)), can delay the degradation of mouse articular cartilage and the formation of osteophytes in OA 9. Runx1 promotes the differentiation of chondrocytes into osteoblasts and further accelerates bone formation 18. The Cbfβ subunit is a non-DNA-binding protein that binds Cbfα proteins to mediate their DNA-binding affinities 10, 18. During postnatal skeletal development, Cbfβ mediated chondrocyte maturation is pivotal for trabecular bone morphogenesis 13. Further, Guofeng Li *et al.* and Guangdi Li *et al.* found that Cbfβ is the one of potential key transcriptional factors in human cartilage and OA 19, 20, and the protein expression of Cbfβ in cartilages of human OA decreased significantly 21. These studies suggest that Cbfβ may play an important role in OA, nevertheless, the function of Cbfβ in OA pathogenesis is still lacking the validation of loss-of-function animal model. A recent study has shown that a small molecule kartogenin promoted chondrocyte differentiation and exhibited chondroprotective effects in OA animal models by upregulating the Cbfβ-Runx1 transcriptional program 22. However, the detailed regulatory mechanism of Cbfβ in OA pathogenesis remains unclear.

Here, we found that the conditional knockout of Cbfβ in mice postnatal articular cartilage resulted in a spontaneous OA phenotype, with a significant increase in the expression of cartilage degradation markers and inflammatory markers in synovium. Moreover, our results indicate that the absence of Cbfβ leads to activation of the Wnt signaling pathway and inhibition of the Yap-TGFβ signaling pathway, leading to cartilage ossification and osteophyte formation, thus aggravating the pathological process of OA in mice. Loss of Cbfβ in cartilage affected the physiological homeostasis of cartilage, thereby exacerbating the entire pathological process of OA due to impaired orchestration of multiple signaling pathways. In summary, our findings demonstrate that Cbfβ is a central regulator orchestrating Wnt/β-catenin, Hippo/Yap, and TGFβ signaling pathways to maintain articular cartilage homeostasis and protect from OA. Elucidating Cbfβ's role in regulating multiple signaling pathways allows us to more fully understand the mechanisms underlying OA pathogenesis and will potentially facilitate the development of novel and effective OA treatments.

## Results

### Tamoxifen (TMX) induced Cbfβ^f/f^Aggrecan-CreER^T^ mice developed spontaneous early-onset OA

**Figure 1:**
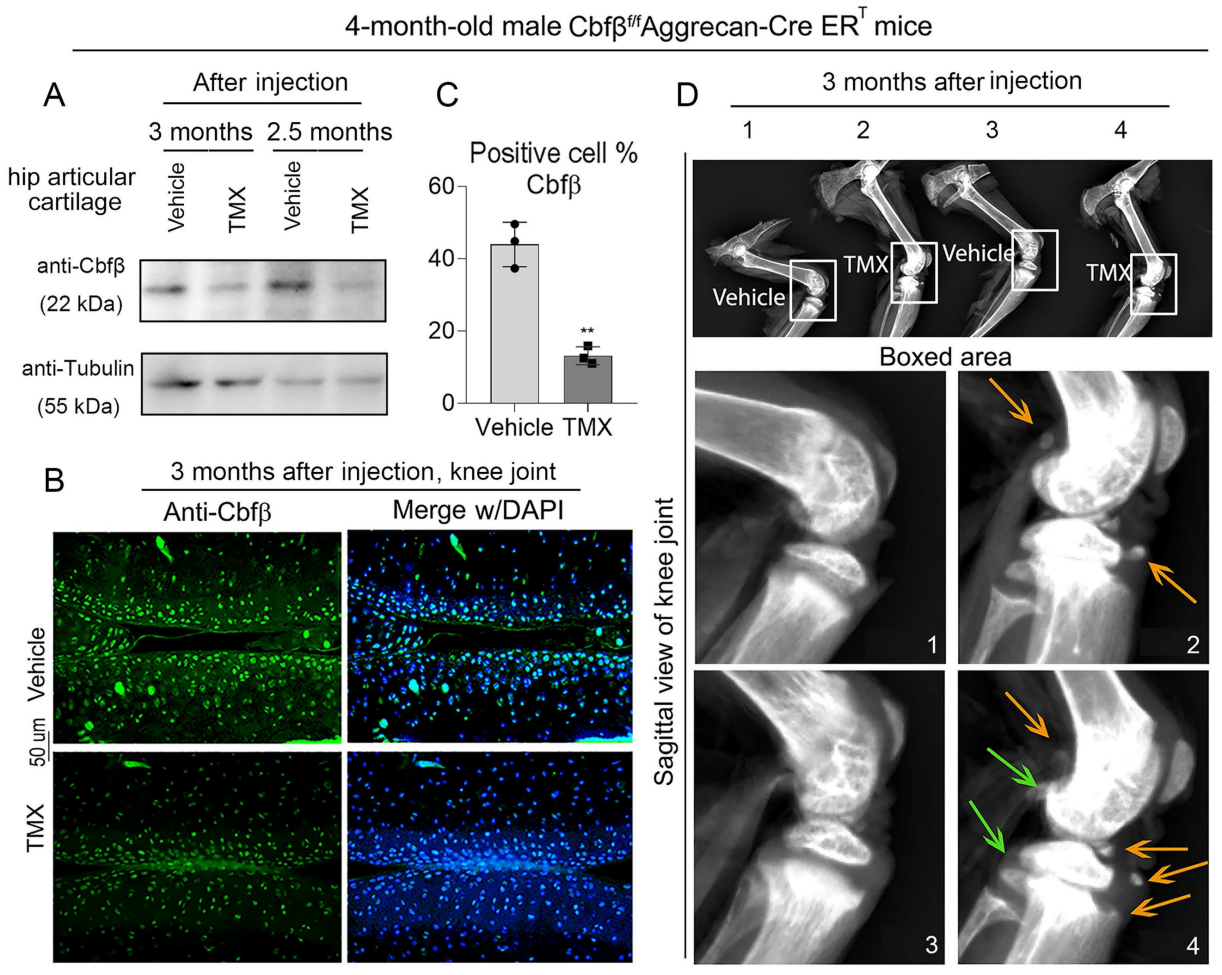
**Cbfβ^f/f^Aggrecan-CreER^T^ tamoxifen (TMX) induced mice developed spontaneous early onset OA. (A)** Western blot to examine Cbfβ protein expression level after TMX or corn oil (Vehicle) injection in 4-month-old male Cbfβ^f/f^Aggrecan-CreER^T^ mice hip articular cartilage. **(B)** Immunofluorescence (IF) staining of Cbfβ in knee joint from 4-month-old Cbfβ^f/f^Aggrecan-CreER^T^ male mice induced by vehicle or TMX. **(C)** Quantification of B, n=3. **(D)** X-ray of 4-month-old Cbfβ^f/f^Aggrecan-CreER^T^ male mice knee. 1 and 3: injected vehicle, 2 and 4: injected TMX. Green arrows show osteophytes and orange arrows show OA-related bone destruction. The results are presented as the mean ± SD. **, *p*<0.01.

To study the role of Cbfβ in spontaneous OA, we generated TMX inducible Cbfβ^f/f^Aggrecan-CreER^T^ mice by crossing Cbfβ^f/f^ mice with TMX inducible Aggrecan-CreER^T^ mice. We first examined the knockout efficiency of Cbfβ in articular cartilage of mice, and western blot results showed that Cbfβ protein levels significantly decreased in articular cartilage tissues from Cbfβ^f/f^Aggrecan-CreER^T^ mice hip joint after TMX injection (**Fig. 1A**). Immunofluorescence (IF) staining against Cbfβ in TMX-injected Cbfβ^f/f^Aggrecan-CreER^T^ mice knee joint articular cartilage also showed a decrease in the percentage of chondrocytes expressing Cbfβ, compared to those in vehicle-treated (control) mice (**Fig. 1B, C**). Those data showed successful Cbfβ knockdown in TMX-induced Cbfβ^f/f^Aggrecan-CreER^T^ mice cartilage. We next investigated the morphological changes of knee joints in Cbfβ deficient mice compared to control vehicle mice. Radiographs showed unclear borders and narrowed joint space in TMX-induced Cbfβ^f/f^Aggrecan-CreER^T^ mice knee joint compared to those of control groups (**Fig. 1D**). In addition, we also observed damaged articular cartilage around the joint, damaged lateral meniscus, as well as abnormal synovium and osteophyte formation in TMX induced Cbfβ conditional knockout (CKO) mice (**Fig. 1D**). These results show that Cbfβ deficient mice develop spontaneous early onset OA phenotypes, indicating that Cbfβ plays a key role in OA-associated joint destruction.

### Cbfβ deletion in articular cartilage resulted in an OA-like phenotype with decreased Aggrecan and Col2α1 expression *in vivo*

**Figure 2:**
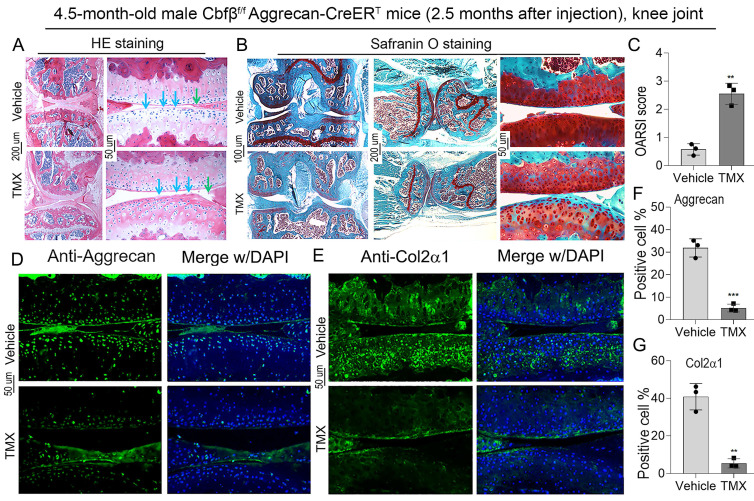
** Deletion of Cbfβ in articular cartilage resulted in an OA-like phenotype with decreased Aggrecan and Col2α1 expression *in vivo*. (A)** H&E staining and **(B)** Safranin O staining of the knee joint from TMX or vehicle induction in 4.5-month-old male Cbfβ^f/f^Aggrecan-CreER^T^ mice. Blue and green arrows show irregular knee joint surface in TMX-induced Cbfβ^f/f^Aggrecan-CreER^T^ mice. **(C)** Knee joint Osteoarthritis Research Society International (OARSI) score of B, n=3. **(D, E)** IF staining of Aggrecan and Col2α1 in knee joint from 4.5-month-old male Cbfβ^f/f^Aggrecan-CreER^T^ mice. **(F, G)** Quantification of D and E, n=3. The results are presented as the mean ± SD. **, *p*<0.01; ***, *p*<0.001.

To further explore the role of Cbfβ in articular cartilage, we carried out the histological analysis of knee joints in Cbfβ^f/f^Aggrecan-CreER^T^ mice induced by either vehicle or TMX. Hematoxylin and eosin (H&E) staining showed irregularity on the articular cartilage surface of TMX induced group, suggesting increased joint wear (**Fig. 2A**). Safranin O (SO) staining demonstrated degraded articular cartilage with increased subchondral bone volume in 4.5-month-old TMX induced Cbfβ^f/f^Aggrecan-CreER^T^ mice (**Fig. 2B**). Specifically, SO staining of the knee joints showed that TMX induced Cbfβ-deficient mice had disturbed cartilage surface, decreased joint space, and disorganized cartilage in the synovium, similar to morphology of injured knee joints (**Fig. 2B**). These findings were reflected in increased Osteoarthritis Research Society International (OARSI) scores of TMX induced Cbfβ-deficient mice (**Fig. 2C**). In addition, we detected the marker expression of chondrogenesis by IF staining and found that Aggrecan and Col2α1 expression was significantly reduced in the articular cartilage of Cbfβ-deficient mice (**Fig. 2D-G**). These data reveal that Cbfβ plays an important role in maintaining postnatal articular cartilage in OA onset and progression.

### The absence of Cbfβ in articular cartilage leads to increased articular cartilage degradation markers expression and inflammatory infiltration *in vivo*

**Figure 3:**
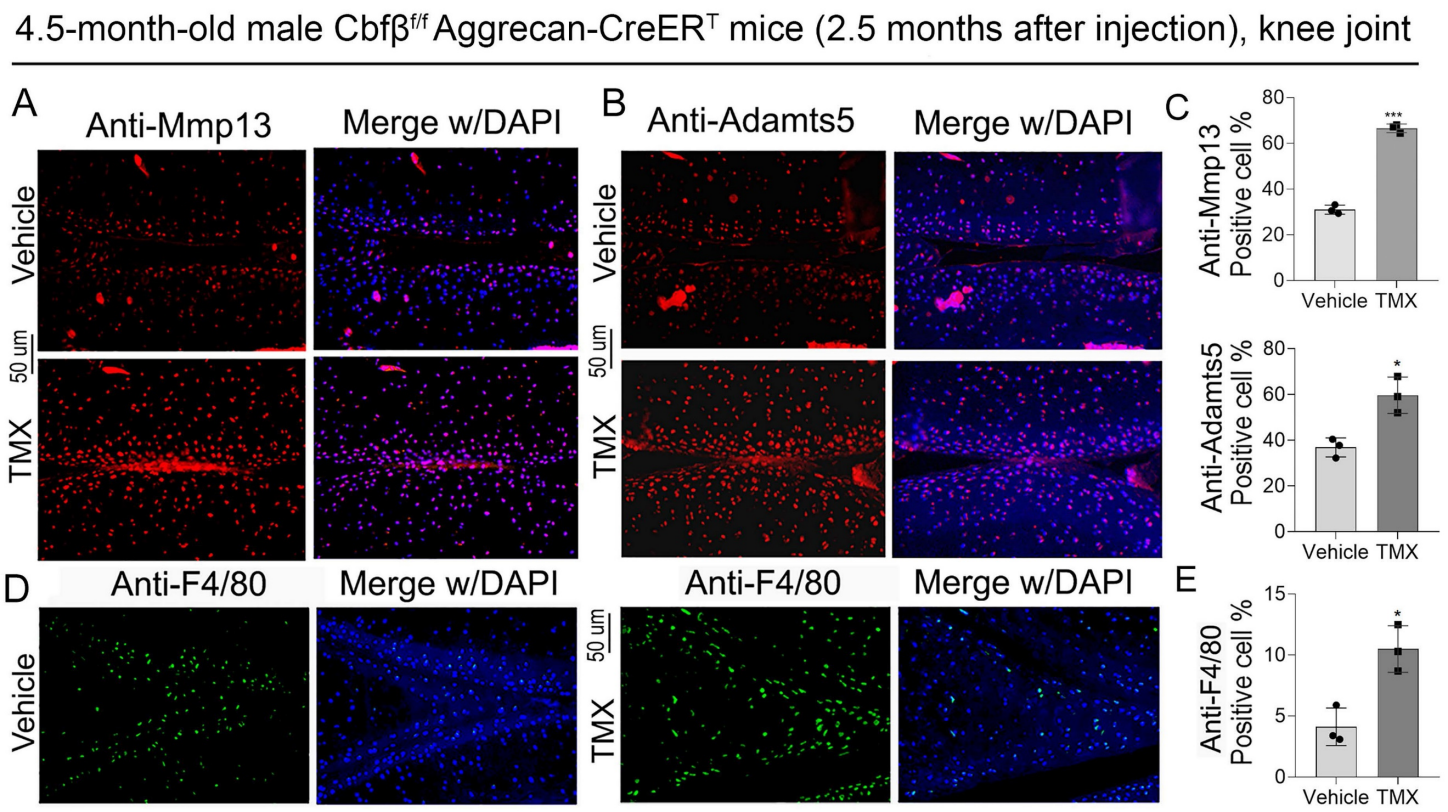
**Deletion of Cbfβ in articular cartilage increased articular cartilage degradation markers *in vivo*. (A, B)** IF staining of Mmp13 and Adamts5 in knee joint from 4.5-month-old male Cbfβ^f/f^Aggrecan-CreER^T^ mice with TMX or vehicle induction. **(C)** Quantification of A and B, n=3.** (D)** IF staining of F4/80 in the synovium of the knee joint from 4.5-month-old male Cbfβ^f/f^Aggrecan-CreER^T^ mice. **(E)** Quantification of D, n=3. The results are presented as the mean ± SD. *, *p*<0.05; ***, *p*<0.001.

ADAMTSs and MMPs, especially Adamts5 and Mmp13, play crucial roles in cartilage destruction in OA 23, 24. We found that in the knee joint of Cbfβ conditional knockout mice, the expression of articular cartilage degradation markers Mmp13 and Adamts5 significantly upregulated in chondrocytes as shown by IF staining, indicating increased degradation in Cbfβ-deficient mice articular cartilage (**Fig. 3A-C**). Furthermore, in OA mice, cartilage degradation in the joint is often accompanied by increased inflammatory infiltration in the synovial region, especially with a massive infiltration of F4/80^+^ macrophages, which leads to joint enlargement 25-27. Therefore, we further detected the expression of immune cell markers in the knee synovium. IF staining results showed that knee synovium of TMX induced Cbfβ CKO mice had increased F4/80^+^ macrophages when compared to vehicle mice (**Fig. 3D, E**), suggesting that lack of Cbfβ in articular cartilage exacerbated inflammatory response in the joint synovium of OA mice. Collectively, our data showed that articular cartilage-specific Cbfβ deletion in adult mice led to an OA-like phenotype with severely degraded articular cartilage and increased inflammatory infiltration in the knee joint synovium.

### Cbfβ protected against articular cartilage destruction in OA by orchestrating multiple signal pathways

**Figure 4:**
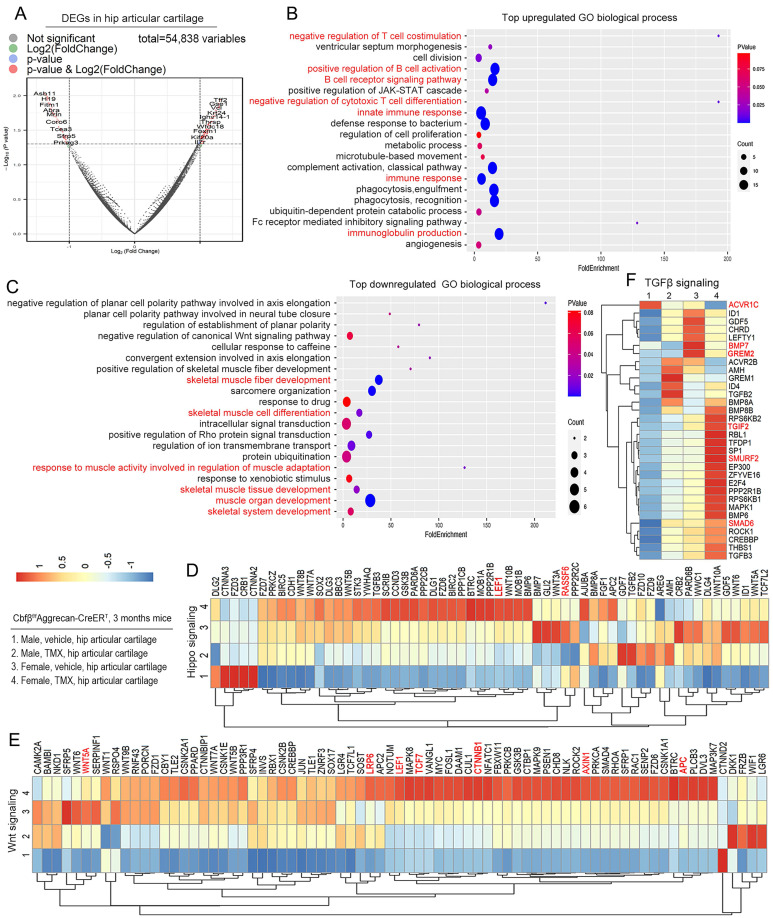
**RNA-sequencing analysis of Cbfβ-deficient mice hip articular cartilage shows altered TGFβ, Wnt, and Hippo signaling pathways. (A)** The volcano plot showed differentially expressed genes (DEGs). Hip articular cartilages from 3-month-old female Cbfβ^f/f^Aggrecan-CreER^T^ mice induced by vehicle or TMX. **(B-C)** Gene Ontology (GO) functional clustering of the top upregulated biological process (BP) in hip articular cartilages from 3-month-old female Cbfβ-deficient mice.** (C)** GO functional clustering of the top downregulated BP. **(D)** Heatmap for Hippo signaling related gene expression in hip articular cartilage of 3-month-old Cbfβ^f/f^Aggrecan-CreER^T^ mice. (1) Male, Vehicle, hip articular cartilage, (2) Male, TMX, hip articular cartilage, (3) Female, Vehicle, hip articular cartilage, (4) Female, TMX, hip articular cartilage. **(E)** Heatmap for Wnt signaling-related gene expression in hip articular cartilage of 3-month-old Cbfβ^f/f^Aggrecan-CreER^T^ mice. **(F)** Heatmap for TGFβ signaling-related gene expression in hip articular cartilage of 3-month-old Cbfβ^f/f^Aggrecan-CreER^T^ mice.

We further investigated the mechanism underlying the role of Cbfβ in OA at the transcriptional level by performing RNA-sequencing analysis of Cbfβ^f/f^Aggrecan-CreER^T^ hip articular cartilage to explore Cbfβ downstream target genes and related signaling pathways. Significant Differentially expressed genes (DEGs) (*p*<0.05) were found in the hip articular cartilage of Cbfβ^f/f^Aggrecan-CreER^T^ mice treated with TMX or vehicle (oil) control, with 62.4% genes up-regulated and 37.6% genes down-regulated in TMX induced Cbfβ CKO mice, indicating that Cbfβ is an important transcription regulatory factor in maintaining joint stability (**Fig. S1A**). In the hip articular cartilage samples, volcano plot showed top upregulated DEGs includes Tff2, Ighv5-6, Notch1, Plin4, Retn, Foxm1, Angptl4, Tmem131, and Il7r, while downregulated DEGs includes Asb11, Srl, Fitm1, Tmem52, Asb2, Mrln, Prss23, Clec3b, Tcea3, Sfrp2, Sfrp5, and Asb15 (**Fig. 4A**, **Fig. S1B-C**). Sfrp5 is an antagonist that directly blocks Wnt signaling by binding to Fz protein or forming a nonfunctional complex with Fz 28. Volcano plot showed that the expression level of Sfrp5 in articular cartilage with Cbfβ deficiency is significantly reduced in TMX-induced Cbfβ CKO mice (**Fig. 4A**, **Fig. S1B**), indicating that Cbfβ CKO mice have relieved blocking in Wnt signaling. Moreover, transcription extension factor Tcea3 binds to TGFβ receptor I to activate the TGFβ signaling pathway and regulate the phosphorylation level of Smad2 29. We observed significantly reduced expression of Tcea3 in Cbfβ-deficient mice articular cartilages, which may lead to an inhibited TGFβ signaling pathway (**Fig. 4A**, **Fig. S1B**). We also found that the Med12 gene expression significantly increased in articular cartilage lacking Cbfβ (**Fig. S1C**). Studies have shown that interfering with the Med12 gene reduces the expression of Wnt4 and β-catenin 30, and activates the TGFβ pathway 31, suggesting that Cbfβ may be involved in regulating both Wnt/β-catenin and TGFβ signaling pathways.

To further understand the regulatory function of Cbfβ in articular cartilage, Gene Ontology (GO) analysis was performed on the DEGs we found (**Fig. 4B, C**). Evaluation of the significant GO Biological Process (BP) in mice with TMX induction showed enhanced innate immune response, immune response, immunoglobulin production, positive regulation of B cell activation, B cell receptor signaling pathway, negative regulation of T cell co-stimulation and negative regulation of cytotoxic T cell differentiation (**Fig. 4B**). These signaling pathway changes suggested that Cbfβ deletion exacerbated multiple immune signaling pathways in hip joint, indicated that Cbfβ has a vital role in controlling the immune response in OA pathogenesis.

Moreover, the downregulated GO biological process enriched in the positive regulation of skeletal muscle fiber development, skeletal muscle fiber development, skeletal muscle cell differentiation, skeletal muscle tissue development, muscle organ development, and skeletal system development in the hips of Cbfβ-deficient mice (**Fig. 4C**). It is worth noting that the joint pain and decreased mobility caused by OA lead to uncoordinated muscle movements, atrophy, and weakness of the muscles around the affected joints 3. Our findings indicated that loss of Cbfβ in articular cartilage affected the physiological homeostasis of surrounding tissues, such as bone tissue, synovium, and muscles, thereby exacerbating the entire pathological process of OA.

Wnt, Hippo, and TGFβ signaling pathways play vital roles in maintaining joint stability in OA 9. Through GO biological process analysis, we found that negative regulation of the canonical Wnt signaling pathway was down-regulated in Cbfβ-deficient hip tissue, indicating that Cbfβ deletion in mice activates canonical Wnt signaling pathway and leads to osteophyte formation and cartilage ossification in mice joint (**Fig. 4C**). We further analyzed the expression level of genes in Hippo, Wnt, and TGFβ signaling at individual level through heatmap analysis (**Fig. 4D-F**). Our results showed that some Hippo signaling genes were downregulated in Cbfβ-deficient hip articular cartilage such as the Rassf6 (**Fig. 4D**). When Rassf6 combines with Mst2, Rassf6 inhibits Mst2 activity and antagonizes Hippo signaling 32. These results suggested that loss of Cbfβ could result in the activation of the Hippo signaling pathway in the hip articular cartilage. Ctnnb1 encoded β-catenin protein is a key factor in the canonical Wnt signaling pathway and is also regulated by Hippo signaling 9. We found that genes associated with Wnt signaling, such as Ctnnb1, Lrp6, Axin1, APC, Tcf7, and Lef1, were significantly upregulated in Cbfβ-deficient hips (**Fig. 4E**), indicating that loss of Cbfβ could promote cartilage ossification and osteophyte formation through its regulation of the Wnt signaling pathway.

In addition, TGFβ signaling pathway repressors including the Tgif2 33, Smad6 34, and Smurf2 35 were upregulated in the Cbfβ-deficient hip articular cartilage, while other critical genes in the TGFβ signaling pathway, such as Acvrl1, Bmp7, and Grem2, were down-regulated in Cbfβ-deficient hip joints (**Fig. 4F**). Collectively, these data suggest that loss of Cbfβ in hip articular cartilage inhibits the expression of critical genes in the TGFβ signaling pathway and promote the expression of TGFβ signaling repressors, resulting in TGFβ signaling pathway inhibition. Together, those results showed that Cbfβ deficiency leads to upregulated Hippo and Wnt signaling pathways, and downregulated TGFβ signaling pathways in mice hip articular cartilage, indicating its crucial regulatory role in all those mentioned signaling pathways.

To further investigate to assess sex-specific regulatory functions of Cbfβ, we also compared the gene expression profile of Cbfβ-deficient male and female hip articular cartilage tissues (**Fig. 4D-F**). We found that most of the DEGs in Wnt/β-catenin, Hippo/Yap, and TGFβ signaling pathways showed similar changing trends in male and female Cbfβ-deficient mice compared to control vehicle induced mice, but there were also sex differences (**Fig. 4D-F**). The changes in gene expression were more significant in female TMX-induced Cbfβ-deficiency mice compared to male mice (**Fig. 4D-F**). This may also be consistent with the higher incidence of bone and joint in female patients in clinical patients. Collectively, RNA-sequencing analysis demonstrated the role of Cbfβ as a central regulator in the canonical Wnt, Hippo, and TGFβ signaling pathways, thereby exacerbating a series of OA pathological processes, including cartilage damage, inflammation, muscular atrophy, and bone ossification.

### Cbfβ orchestrated signals of Hippo/Yap, TGFβ/Smad, and Wnt/β-catenin in articular cartilage

**Figure 5:**
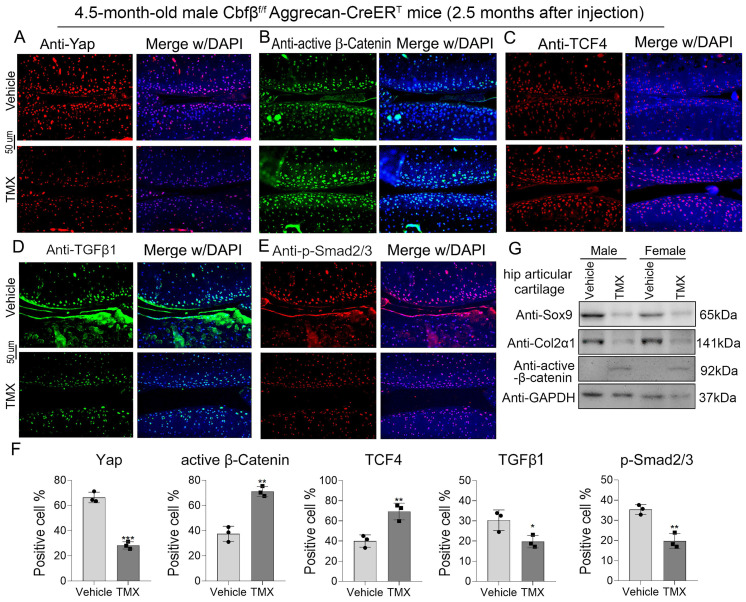
** Cbfβ deletion in articular cartilage resulted in altered key genes in Hippo/Yap, TGFβ/Smad, and Wnt/β-catenin signaling pathways *in vivo*. (A-E)** IF staining of Yap, active β-Catenin, TCF4, TGFβ1, and p-Smad2/3 in knee articular cartilage from 4.5-month-old male Cbfβ^f/f^Aggrecan-CreER^T^ mice induced by vehicle or TMX. **(F)** Quantification data analysis of A-E, n=3. **(G)** Western blot data show the protein levels changes of Sox9, Col2α1, and active β-Catenin in male and female mice hip articular cartilages. The results are presented as the mean ± SD. *, *p*<0.05; **, *p*<0.01; ***, *p*<0.001.

**Figure 6:**
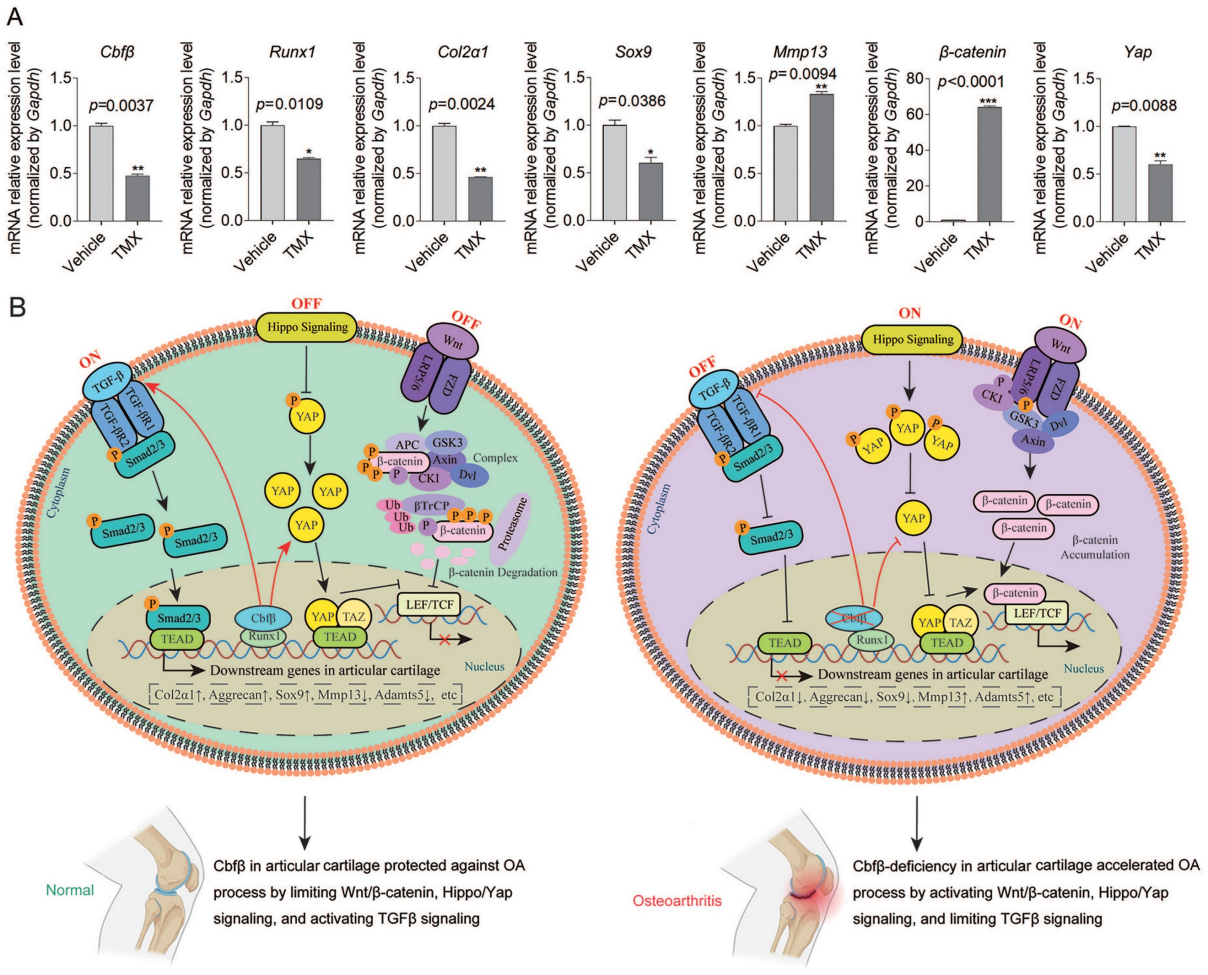
** Cbfβ orchestrated Hippo/Yap, TGFβ/Smad, and Wnt/β-catenin signaling pathways in articular cartilage. (A)** qPCR data show the mRNA expression levels changes of Cbfβ, Runx1, Col2α1, Sox9, Mmp13, β-catenin, and Yap in hip articular cartilages from Cbfβ^f/f^Aggrecan-CreER^T^ mice induced by vehicle or TMX. *, *p*<0.05; **, *p*<0.01; ***, *p*<0.001. n=3 **(B)** Working model of Cbfβ orchestrating Wnt/β-catenin Hippo/Yap and TGFβ signaling of chondrocytes in articular cartilage in normal vs Cbfβ-deficient OA phenotype joints.

We sought to further confirm Cbfβ's regulatory mechanism in the canonical Wnt, Hippo, and TGFβ signaling pathways at the transcriptional and translational level by detecting the mRNA and protein expression levels of key genes in these signaling pathways (**Fig. 5, Fig. 6A**). Through IF staining, we found that Cbfβ deletion in articular cartilage was associated with a decreased percentage of cells expressing Yap (**Fig. 5A**), TGFβ1 (**Fig. 5D**), and p-Smad2/3 (**Fig. 5E**), while increased percentage of cells expressing active β-Catenin (**Fig. 5B**) and TCF4 (**Fig. 5C**) in the cartilage of the knee joint. Quantitative analysis of these IF staining results further supported these conclusions (**Fig. 5F**). Moreover, western blot results showed that the absence of Cbfβ led to a decrease in Sox9 and Col2α1 expression and an increase of active β-Catenin expression in hip articular cartilage (**Fig. 5G**). Furthermore, we implemented qPCR to verify the mRNA expression of these pathway-related genes. Our results confirmed the knockdown efficiency of Cbfβ mRNA level in Cbfβ^f/f^Aggrecan-CreER^T^ mice hip articular cartilage tissue after induction by TMX. We also found that conditional deletion of Cbfβ with the decreased mRNA levels of Runx1, Col2α1, Sox9, and Yap, along with an increase in expression of Mmp13 and β-Catenin (Ctnnb1) (**Fig. 6A**). Altogether, our data demonstrated that Cbfβ could be a key scaffolding in protecting against OA process via controlling Wnt/β-Catenin, Hippo and TGFβ signaling pathways (**Fig. 6B**), and indicated that Cbfβ plays a key role in maintaining cartilage homeostasis.

## Discussion

In the present study, we demonstrated that conditional knockout of Cbfβ in postnatal mice articular cartilage caused severe spontaneous OA, such as damaged cartilage, bone hyperosteogeny, and inflammatory response. Further analysis revealed that Cbfβ modulated articular cartilage regeneration by regulating multiple key signaling pathways including Wnt/β-catenin, TGFβ, and Hippo/Yap.

Currently, the treatment options for OA are limited to managing pain and undergoing surgery, which is a significant concern for the aging population 2, 36. There are many candidate genes involved in the damage of the articular cartilage and subchondral bone in OA pathogenesis, including Sox9, Adamts5, Mmp13, Yap, Wnt/β-catenin signaling, and TGFβ signaling 4, 37-42. However, the root causes of the disease remain unclear. A recent study has identified that Cbfβ may play an important role in articular cartilage regeneration and repair in OA 21. The results presented in our study support this hypothesis, as we found that the expression of Cbfβ dramatically impacts the integrity of mouse cartilage, and the conditional knockout of Cbfβ leads to the development of spontaneous OA-like phenotype characterized by cartilage degradation and subchondral bone intrusion. Additionally, Col2α1 and Aggrecan are crucial in maintaining the cartilaginous matrix and proper structure and function of cartilage 43. Our findings also showed that postnatal deletion of Cbfβ led to a significant reduction in Col2α1 and Aggrecan expression in cartilage, which suggests that the modulation of these critical proteins by Cbfβ is a key component in the progression of OA.

In both clinical data and animal models, the canonical Wnt signaling pathway has been implicated in OA 37, 44. However, the mechanism underlying how Wnt canonical signaling is dysregulated in OA is unclear. Here, we revealed that deficiency of Cbfβ impairs cartilage homeostasis by orchestrating multiple signaling pathways, including Wnt/β-catenin. Specifically, our data has indicated that conditional knockout of Cbfβ in Cbfβ^f/f^Aggrecan-CreER^T^ mouse models resulted in the increased expression of active β-catenin, along with the previously reported downstream effector of canonical Wnt-signaling, TCF4 45. Our RNA-seq also showed that Ctnnb1, Lrp6, Axin1, APC, Tcf7, and Lef1 were significantly increased in Cbfβ-deficient hips. Ctnnb1 gene encoded β-catenin protein is a key functional effector molecule downstream of the canonical Wnt signaling pathway, crucial in bone homeostasis regulation 44, 46. Moreover, the Di Chen lab has generated β-catenin(ex3)^f/f^Col2α1-CreER^T^ mice and has found that the inhibition of β-catenin protein levels in the nucleus of articular chondrocytes could limit the exacerbation of OA-like phenotypes, including articular cartilage damage and osteophyte formation 47. They also found similar results in another animal model β-catenin(ex3)^f/f^Agc1-CreER^T^, in which the activation of β-catenin in Aggrecan-expressing cells in joint exacerbated OA progression, fully indicating that the expression of β-catenin in the nucleus is essential for maintaining joint homeostasis 48. Here, we found that Cbfβ deficiency may lead to excessive activation of the Wnt pathway, and the highly expressed β-catenin would not be degraded and accumulate in the nucleus, further promoting the activation of β-catenin as a co-activator of TCF/LEF family transcription factors. This may be one of the important reasons for the formation of osteophytes and cartilage ossification in Cbfβ deficient mice.

Previously, we found that Cbfβ and Runx1 control osteoblast-adipocyte lineage interaction by β-catenin signaling, and positively regulate active β-catenin expression in MSC progenitor cells and osteoblasts 11. The regulation of the Wnt/β-catenin pathway by Cbfβ may be different in the mechanism of bone formation and OA cartilage defects, thus the mechanism may need to be further explored to better understand this overlap. On the one hand, we speculate that this difference might be caused by the change of Yap expression in cartilage joints. It is worth noting that the Hippo/Yap pathway is closely related to the Wnt/β-catenin pathway 49, 50. Yap is a key regulatory molecule in the Hippo signaling pathway, and Yap activity is necessary for tissue regeneration after tissue injury 51. When the Hippo signaling pathway is turned off, Yap/Taz is not phosphorylated and successfully enters the nucleus to form complexes with the transcription factor TEADs, further regulating gene transcription to influence multiple steps of chondrocyte differentiation including the upregulation of chondroprogenitor cell proliferation and the inhibition of chondrocyte maturation 52. Moreover, the regulation of β-catenin activity by Yap has received extensive attention 53, 54, Yap can affect the ubiquitination of β-catenin, then regulate the degradation of β-Catenin through the proteasome pathway, and β-catenin cannot enter the nucleus to play its role as a co-transcription factor 9, 53, 54. We found that the expression of Yap in the articular cartilage of Cbfβ postnatal deletion mice was significantly decreased, and the inhibitory effect of Yap on β-catenin activity was weakened, which may result in a significant increase in the expression of active β-catenin in Cbfβ^f/f^Aggrecan-CreER^T^ mice articular cartilage. Our data also supports that Cbfβ may suppress the Hippo/Yap pathway and the Wnt/β-catenin pathway in OA articular cartilage homeostasis. On the other hand, Cbfβ combines the Runx family proteins in regulating skeletal changes, including Runx1, Runx2, and Runx3 10, 55. We recently reported that Runx1 is a critical regulator of articular cartilage homeostasis 18 due in part to modulation of canonical Wnt-signaling provides further vindication of these results and suggests that this interaction between Runx1 and Cbfβ is required to exert their transcriptional effect 9, 56. These results are similar to our previous study on Runx1 9, 10, we suspect that Cbfβ may promote Yap expression by regulating Runx1 expression and Cbfβ/Runx1-Yap protein-protein interaction, and also suggests that the combination of Cbfβ and Runx1 plays a similar role in delaying the pathological process of OA, but further study is needed. Overall, the data presented in this paper sheds further light on how Wnt canonical signaling and Hippo/Yap signaling pathway are regulated by Cbfβ during OA pathogenesis, which may lead to novel therapies for the treatment of this degenerative disease.

Moreover, different from Runx1-deficient mice, the downregulated GO biological process of Cbfβ-deficient hips observably enriched in the skeletal muscle fiber development, skeletal muscle cell differentiation, skeletal muscle tissue development, and muscle organ development. These processes are closely related to OA joint muscle atrophy and dysregulation 3. OA is not just a disease of the cartilage but involves the entire joint (synovial membrane, subchondral bone, muscle, joint capsule), and the wear and tear of the cartilage also causes uncoordinated muscle movements, atrophy, and weakness of the muscles around the affected joints. Furthermore, we also found that lack of Cbfβ exacerbated inflammatory infiltration of the joint synovium. These results suggest that the loss of Cbfβ in cartilage not only affects the cartilage tissue but also changes the physiological homeostasis of surrounding tissues such as muscles and synovium. It also fully reflects the important function of Cbfβ in OA, and that OA is a complex disease with multiple tissue disorders.

Additionally, our study also demonstrated the critical role TGFβ/Smad signaling plays in the spontaneous development of the OA phenotype. The physiological maintenance of articular cartilage requires tight control of the TGFβ signaling pathway 57. The TGFβ signaling pathway may have different roles in physiological and pathological processes 58. The gene expression encoding the TGF-β subtype is closely related to the normal development of cartilage 58. Smad3 knockout mice showed spontaneous joint degeneration, and decreased TGFβ signal transduction and p-Smad3 levels were consistent with pathological changes in OA 58. Previous work has demonstrated the critical role of Cbfβ and Runx1 as mediators of TGFβ signaling 9, 59, with TGFβ signaling activation having been shown to increase Cbfβ and Runx1 expression, while the deletion of Cbfβ attenuated TGFβ signaling pathway activation 21. Our results are consistent and show that conditional knockout of Cbfβ expression significantly reduced cartilage cells expressing TGFβ1. The disruption of TGFβ signaling by deletion of Cbfβ in the articular cartilage showed an increase in catabolic cytokines and enzymes such as Mmp9, Mmp13, Mmp14, Mmp15, IL-6, IL-17, IL-18, and IL-22 21. Our results also indicated that the conditional lack of Cbfβ resulted in significantly elevated expression of Mmp13, suggesting a possible therapeutic target for preventing or reducing OA progression. During the signal activation process of the TGFβ signaling pathway, Smad proteins must be phosphorylated to facilitate the transcription of bone and cartilage homeostasis mediators 60. Notably, our IF staining results showed that conditional knockout of Cbfβ expression resulted in a significant reduction of p-Smad2/3 in articular cartilage, and Jie Shen *et al.* also found that p-Smad3 expression was sensibly decreased in the mouse model of OA by immunohistochemistry 61. However, Xiangguo Che *et al.* found that p-Smad3 expression increased in Cbfβ-deficient mice 21. This difference may be caused by the use of different mouse models, as they crossed Gdf5-Cre transgenic mice with Cbfβ^f/f^ to conditional knockout Cbfβ, and Cbfb^f/f^ served as wild-type control 21. In our study, we used Cbfβ^f/f^ mice with the inducible Aggrecan-CreER^T^ mice to generate Cbfβ^f/f^ Aggrecan-CreER^T^ mice, and adult Cbfβ^f/f^ Aggrecan-CreER^T^ mice received TMX injection to generate postnatal Cbfβ deletion mice, and vehicle-injected Cbfβ^f/f^Aggrecan-CreER^T^ mice as control mice. In addition, the age of the mice may also contribute to the difference. Xiangguo Che *et al.* used 20-week-old mice 21, and we used mice 2.5 months after TMX injection, which is 4.5 months of actual age, which may also be somewhat different. Thus, the mechanism by which Cbfβ expression affects p-Smad2/3 requires further elucidation, as it is not clear whether this function is by either a positive or negative feedback mechanism. In addition, for other key proteins such as Col2α1 and Mmp13, our research results are completely consistent with Xiangguo Che *et al.* 21. These studies fully demonstrate that Cbfβ regulates the cartilage by influencing the TGFβ/Smad signaling pathway. There are still several limitations, as our study did not use the over-expression mouse model to explore the importance of Cbfβ in the prevention of OA, nor did it directly evaluate the effect of Cbfβ deletion on human OA, etc. In future studies, we will continue to explore the specific regulatory mechanisms of Cbfβ on downstream pathways. Specifically, we plan to analyze the promoter sequence of target genes specifically bound by transcription factor Cbfβ, to prove the direct binding and transcriptional regulation function of Cbfβ on downstream target genes.

In summary, we found that Cbfβ deletion in postnatal articular cartilage may not only cause cartilage defect, cartilage ossification, and osteophyte formation but also may be related to inflammatory infiltration of synovium and joint muscle disorder. Our findings suggest that Cbfβ can be a scaffold in maintaining joint homeostasis by orchestrating canonical Wnt/β-catenin, Hippo/Yap, and TGFβ signaling pathways in the pathological process of OA. The novel mechanism provides us with more insights into OA pathogenesis and elucidates possible viable opportunities for therapeutic intervention.

## Materials and Methods

**Generation of Cbfβ inducible conditional deficiency mice.** The Cbfβ^f/f^ mouse line (Stock No: 008765), and Aggrecan-CreER^T^ (Stock No: 019148) were purchased from The Jackson Laboratory. Cbfβ^f/f^ mice were crossed with the inducible Aggrecan-CreER^T^ mice to generate Cbfβ^f/f^Aggrecan-CreER^T^ mice. The genotypes of the mice were determined by polymerase chain reaction (PCR). All mice were maintained under a 12-hour light-dark cycle with ad libitum access to regular food and water at the University of Alabama at Birmingham (UAB) Animal Facility. Tamoxifen (TMX) (T5648, Sigma) was dissolved in vehicle-corn oil (C8267, Sigma) in a concentration of 10 mg/mL and vortexed until clear. The solution was aliquoted and stored at -20^o^C. Before use, the TMX solution was warmed up at room temperature for 15 minutes. Cbfβ^f/f^ Aggrecan-CreER^T^ mice received TMX or vehicle by intraperitoneal (I.P.) injection continuously for 5 days (1 mg per day).

**Radiography.** Radiographs of inducible Cbfβ^f/f^ Aggrecan-CreER^T^ mice were detected by the Faxitron Model MX-20 at 26 kV in the UAB Small Animal Bone Phenotyping Core associated with the Center for Metabolic Bone Disease.

**Histology and tissue preparation.** Histology and tissue preparation was performed as described previously 62. Briefly, mice were euthanized, skinned, and fixed in 4% paraformaldehyde overnight. Joint samples were then dehydrated in ethanol solution and decalcified in 10% EDTA. For paraffin sections, samples were dehydrated in ethanol, cleared in xylene, embedded in paraffin, sectioned at 5 μm with a Leica microtome, and mounted on frosted microscope slides. Hematoxylin and eosin (H&E) and Safranin O (SO)staining were performed as described previously described 9.

**Immunofluorescence (IF) staining.** We used the following primary antibodies: mouse-anti-Cbfβ (1:200, Santa Cruz, sc-56751), mouse-anti-Col2α1 (1:150, Santa Cruz, sc-52658), rabbit-anti-Sox9 (1:200, Santa Cruz, sc-20095), rabbit-anti-Mmp13 (1:200, Abcam, ab39012), rabbit-anti-Adamts5 (1:200, Santa Cruz, sc-83186), rabbit-anti-Yap (1:200, Cell Signaling Technology, 14074 S), mouse-anti-TGFβ1 (1:200, Santa Cruz, sc-130348), rabbit-p-Smad2/3 (1:150, Cell Signaling Technology, 8828S), mouse-anti-TCF4 (1:200, Santa Cruz, sc-166699) and mouse-anti-Active-β-catenin (1:150, Millipore, 05-665). Immune cell markers were detected by using the following primary antibodies: mouse-anti-F4/80 (1:200, Santa Cruz, sc-377009). IF staining images were taken with the Leica DMLB Microscope and the Leica D3000 fluorescent microscope and were quantified by Image J software.

**RNA sample preparation, RNA-seq, and quantitative reverse transcription PCR (qRT-PCR)**. RNA extraction of mice hip articular cartilage tissues was performed with TRIzol reagent (Fisher, 15596018). RNA-seq of our hip articular cartilage tissues was executed by Admera Health Company (South Plainfield, NJ). Sample quality was assessed by Agilent Bioanalyzer, and high-quality RNA libraries were prepared by using the NEB Next Ultra RNA Library Prep Kit. Libraries were analyzed using Illumina next-generation sequencing and relative quantification was provided by Admera Health. Read counts were subjected to paired differential expression analysis using the R package DESeq2. The volcano plot and heatmap were generated by the R package. GO analysis was executed by the DAVID online tool (https://david.ncifcrf.gov/). Besides, reverse transcription was done with the RevertAid RT Reverse Transcription Kit (Fisher, K1691). Our qPCR was performed with StepOne Real-Time PCR System by using Fast SYBR® Green Master Mix (Life Technology, 4385612), and the manufacturer's instructions were followed. qRT-PCR primer sequences have been listed in **Table S1**.

**Western blot.** The tissue we used for WB was articular cartilage isolated from the hip joint of mice, muscle tissue and bone were removed as much as possible. 20 ug proteins of mice cartilage tissues were loaded on SDS-PAGE and electro-transferred on polyvinylidene fluoride membranes. Primary antibodies as following: mouse-anti-Cbfβ (1:500, Santa Cruz, sc-56751), mouse-anti-β Tubulin (1:750, Santa Cruz, sc-166729), mouse-anti-Sox9 (1:500, Santa Cruz, sc-166505), mouse-anti-active-β-catenin (1:300, Millipore, 05-665), mouse-anti-Col2α1 (1:500, Santa cruz, sc-52658), and mouse-anti-GAPDH (1:1,000, Santa Cruz, sc-365062). Rabbit anti-mouse IgG-HRP (1:2,000, sc-358917) from Santa Cruz was used as the secondary antibody.

**Statistical analysis**. In our study, we compared the differences between TMX and vehicle-injected adult Cbfβ^f/f^ Aggrecan-CreER^T^ mice. Data are presented as mean ± SD (n=3). Statistical significance was assessed using Student's t-test. Values were considered statistically significant at *p*<0.05.

## Supplementary Material

Supplementary figure and table.

## Abbreviations

Cbfβ: Core binding factor subunit β
OA: osteoarthritis
Runx1: Runt-related transcription factor 1
CKO: Conditional knockout
H&E: Hematoxylin and eosin
OARSI: Osteoarthritis Research Society International
qRT-PCR: Quantitative reverse transcription polymerase chain reaction
TMX: Tamoxifen
GAPDH: Glyceraldehyde-3-phosphate dehydrogenase
IF: Immunofluorescence
SO: Safranin O
GO: Gene ontology
BP: Biological process
DEGs: Differentially expressed genes
