# Deficiency of Cbfβ in articular cartilage leads to osteoarthritis-like phenotype through Hippo/Yap, TGFβ, and Wnt/β-catenin signaling pathways: Erratum

**DOI:** 10.7150/ijbs.142878

**Published:** 2026-10-12

**Authors:** Yan Zhang, Huiwen Chen, Jinjin Wu, Abigail McVicar, Yilin Chen, Jiacan Su, Yi-Ping Li, Wei Chen

**Affiliations:** 1 Department of Pathology, University of Alabama at Birmingham, Birmingham, AL 35294, USA.; 2 Key Laboratory of Biomedical Information Engineering of Ministry of Education, Biomedical Informatics and Genomics Center, School of Life Science and Technology, Xi'an Jiaotong University, Shaanxi, Xi'an 710049, P.R. China.; 3 Division in Cellular and Molecular Medicine, Department of Pathology and Laboratory Medicine, Tulane University School of Medicine, Tulane University, New Orleans, 70112, USA.; 4 Institute of Translational Medicine, Shanghai University, Shanghai, P.R. China.

The authors regret that a labeling error for the mouse age occurred in Figure 1B. During the labeling process of the figure, we inadvertently labeled the mouse as 3 months after injection, whereas the correct age should be 4.5 months old (2.5 months after injection). Accordingly, we have revised the figure legend of Figure 1B to reflect the correct age of 4.5 months. The corrected Figure 1B and the figure legend are provided, and all authors have agreed to this correction. This correction does not affect the original scientific findings or the conclusions of the study. We sincerely apologize for any inconvenience this error may have caused.

**Correct figure legend of Figure 1B**. Immunofluorescence (IF) staining of Cbfβ in knee joint from 4.5-month-old Cbfβ^f/f^Aggrecan-CreER^T^ male mice induced by vehicle or TMX.

## Figures and Tables

**Figure 1:**
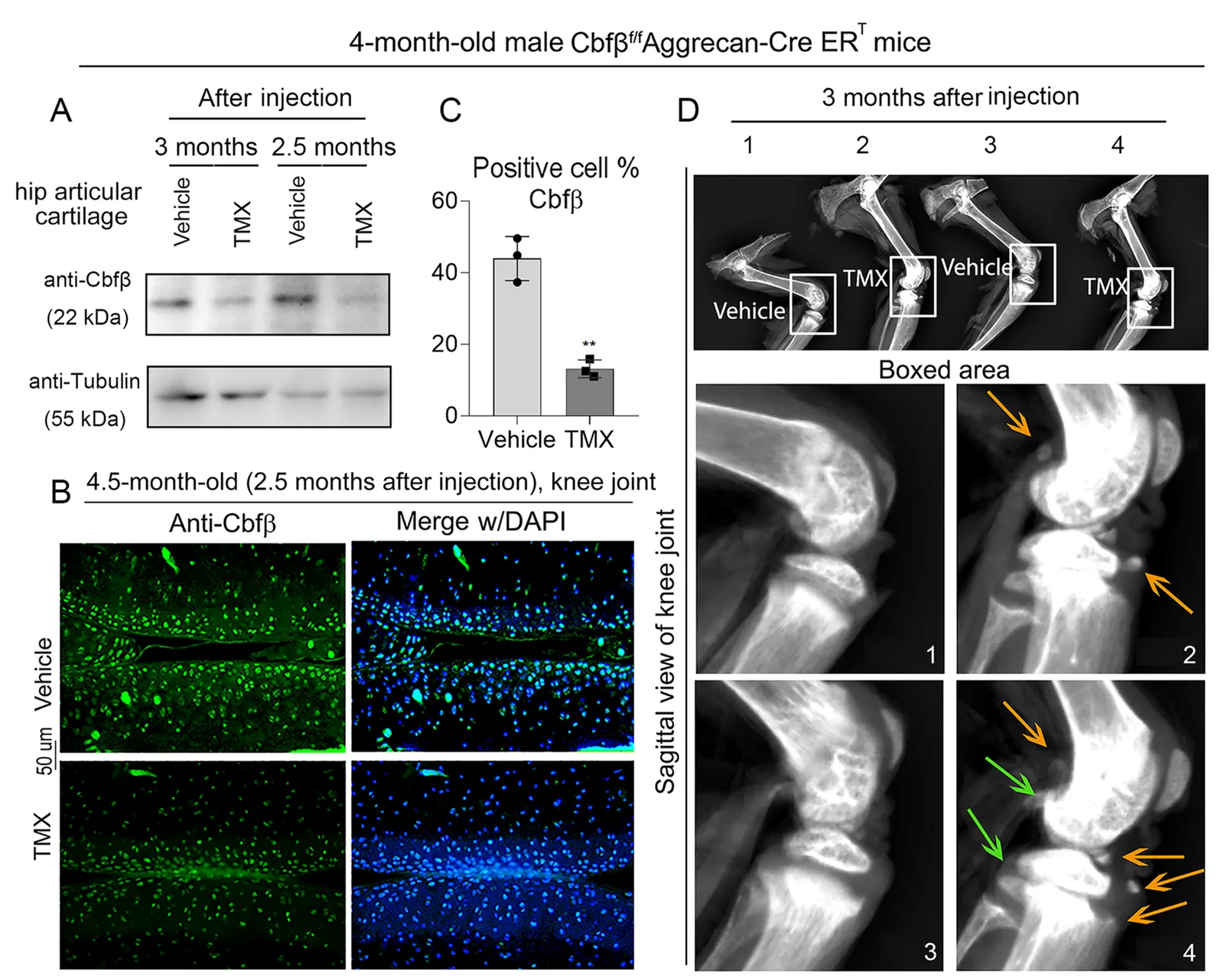
Correct image.

