# Supplementary material for: Deficiency of Cbfβ in articular cartilage leads to osteoarthritis-like phenotype through Hippo/Yap, TGFβ, and Wnt/β-catenin signaling pathways
Source: Int J Biol Sci. 2024 Mar 11;20(6):1965–77. doi: 10.7150/ijbs.90250 (PMC11008268; doi:10.7150/ijbs.90250)
Supplement: Supplementary file 1 — Supplementary figure and table. [file ijbsv20p1965s1.pdf]

Deficiency of Cbfβ in articular cartilage leads to osteoarthritis-like phenotype through Hippo/Yap, TGFβ, and Wnt/β-catenin signaling pathways

Yan Zhang[1, 2], Huiwen Chen[1], Jinjin Wu[1], Abigail McVicar[3], Yilin Chen[3], Jiacan Su[4], Yi-Ping Li[1,3*], Wei Chen[1,3*]

[1] Department of Pathology, University of Alabama at Birmingham, Birmingham, AL 35294, USA.

[2] Key Laboratory of Biomedical Information Engineering of Ministry of Education, Biomedical Informatics and Genomics Center, School of Life Science and Technology, Xi'an Jiaotong University, Shaanxi, Xi'an 710049, P.R. China.

[3] Division in Cellular and Molecular Medicine, Department of Pathology and Laboratory Medicine, Tulane University School of Medicine, Tulane University, New Orleans, 70112, USA.

[4] Institute of Translational Medicine, Shanghai University, Shanghai, P.R. China.

[*] **Corresponding authors:**

Supplemental Figure 1

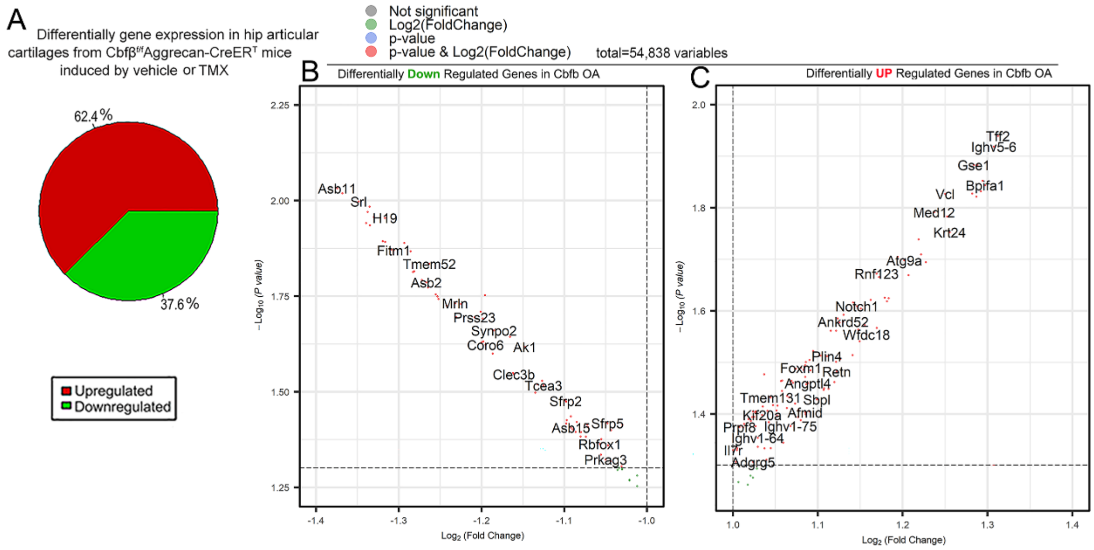

Figure S1. RNA-sequencing found differentially expressed genes (DEGs) in hip articular cartilages from 3-month-old female Cbfβ$^{f/f}$Aggrecan-CreER^{T} mice induced by vehicle and TMX. (A) Pie chart of differentially regulated gene expression in hip articular cartilages. The percentages of genes upregulated and downregulated are shown in red and green, respectively. **(B)** Down DEGs from volcano plot. **(C)** Up DEGs from volcano plot.

Supplemental Table 1

Table S1. qRT-PCR primer sequences

Primer name (Mouse)	Primer sequences (5'-3')
Cbfβ-Forward primer	ACAAACACCTAGCCGGGAATA
Cbfβ-Reverse primer	GCTGTGAAACTCTCACCTCCATT
Runx1-Forward primer	GATGGCACTCTGGTCACCG
Runx1-Reverse primer	GCCGCTCGGAAAAGGACAA
Col2α1-Forward primer	GGGAATGTCCTCTGCGATGAC
Col2α1-Reverse primer	GAAGGGGATCTCGGGGTTG
Sox9-Forward primer	GAGCCGGATCTGAAGAGGGA
Sox9-Reverse primer	GCTTGACGTGTGGCTTGTTC
Mmp13-Forward primer	CTTCTTCTTGTTGAGCTGGACTC
Mmp13-Reverse primer	CTGTGGAGGTCACTGTAGACT
β-catenin (Ctnnb1)-Forward primer	ATGGAGCCGGACAGAAAAGC
β-catenin (Ctnnb1)-Reverse primer	CTTGCCACTCAGGGAAGGA
Yap-Forward primer	ACCCTCGTTTTGCCATGAAC
Yap-Reverse primer	TGTGCTGGGATTGATATTCCGTA
Gapdh-Forward primer	AGGTCGGTGTGAACGGATTTG
Gapdh-Reverse primer	TGTAGACCATGTAGTTGAGGTCA
